# Adiponectin, Leptin, and Leptin Receptor in Obese Patients with Type 2 Diabetes Treated with Insulin Detemir

**DOI:** 10.3390/molecules22081274

**Published:** 2017-07-30

**Authors:** Paweł Olczyk, Robert Koprowski, Katarzyna Komosinska-Vassev, Agnieszka Jura-Półtorak, Katarzyna Winsz-Szczotka, Kornelia Kuźnik-Trocha, Łukasz Mencner, Alicja Telega, Diana Ivanova, Krystyna Olczyk

**Affiliations:** 1 Department of Community Pharmacy, School of Pharmacy and Division of Laboratory Medicine in Sosnowiec, Medical University of Silesia, 41-200 Sosnowiec, Poland; 2 Department of Biomedical Computer Systems, Faculty of Computer Science and Materials Science, Institute of Computer Science, University of Silesia, 41-200 Sosnowiec, Poland; robert.koprowski@us.edu.pl; 3 Department of Clinical Chemistry and Laboratory Diagnostics, School of Pharmacy with the Division of Laboratory Medicine in Sosnowiec, Medical University of Silesia, 41-200 Sosnowiec, Poland; kvassev@sum.edu.pl (K.K.-V.); ajura@sum.edu.pl (A.J.-P.); winsz@sum.edu.pl (K.W.-S.); kkuznik@sum.edu.pl (K.K.-T.); lukasz.mencner@gmail.com (L.M.); alicjatelega@tlen.pl (A.T.); olczyk@sum.edu.pl (K.O.); 4 Department of Biochemistry, Molecular Medicine and Nutrigenomics, The Faculty of Pharmacy, Medical University of Varna, 9002 Varna, Bulgaria; divanova@mu-varna.bg

**Keywords:** diabetes mellitus type 2, adipocytokines, insulin detemir, signal processing, algorithm, Matlab

## Abstract

The aim of the present study is to quantitatively assess the expression of selected regulatory molecules, such as leptin, leptin receptor, and adiponectin in the blood of obese patients with type 2 diabetes both before treatment and after six months of pharmacological therapy with the long-lasting insulin analogue, insulin detemir. A significant decrease in the analysed regulatory molecules, i.e., leptin receptor and adiponectin, was found in blood plasma of the patients with untreated type 2 diabetes. These changes were accompanied by an increase in plasma leptin concentrations. Insulin treatment resulted in the normalization of plasma leptin receptor and adiponectin concentrations. The circulating leptin level did not change following anti-diabetic therapy with insulin detemir. Gender was a significant factor modifying the circulating level of all the analysed regulatory active compounds. Bioinformatic analysis was performed using Matlab with the Signal Processing Toolbox. The conducted discriminant analysis revealed that the leptin receptor, Δw(19), and adiponectin, Δw(21), were the parameters undergoing the most significant quantitative changes during the six-month therapy with insulin detemir. The conducted examinations indicated the contribution of adipocytokines—the biologically-active mediators of systemic metabolism, such as leptin and adiponectin in the pathomechanism of disorders being the basis for obesity which leads to development of insulin resistance, which, in turn, results in the occurrence of type 2 diabetes.

## 1. Introduction

Type 2 diabetes is a disorder resulting from the abnormal secretion or functioning of the key hormone, insulin, which regulates both the biosynthesis of glucose in the liver, as well as glucose utilization by peripheral muscles and fat tissue [1]. In people with advanced type 2 diabetes, the insulin secretion is reduced due to the progressing loss of the secretory function by beta cells of the pancreatic islets. Simultaneously, the response of the peripheral tissues to the activity of the mentioned anabolic hormone is abnormal due to deteriorating insulin sensitivity [1,2]. The main reason for the intensified morbidity of this type of diabetes is the increasing incidence of obesity particularly common in well-developed countries [3]. The excessive body weight gain, which is a significant cause of obesity, is inseparably associated with a chronic, moderately-intensified inflammation which is the consequence of the ‘expansion’ and fat tissue infiltration by the activated macrophage [4,5,6]. Moreover, fat tissue constitutes a source of, among others, 600 active regulatory molecules [7]. Among them, adiponectin, described also as Adipo Q, and leptin play crucial roles in regulating the carbohydrate metabolism, as well as the sensitivity to insulin [7,8,9]. These active molecules may constitute a “bridge” between the fat tissue and metabolic disorders and are most likely to participate in the development of insulin resistance and etiopathogenesis of type 2 diabetes. Recently, the assessment of exogenous insulin influence on the expression of the described regulatory molecules has been a subject of a particular interest as it constitutes the key element of the pharmacotherapy of the disease. A substantial side effect of applying the exogenous insulin is body weight gain [10,11], which is significantly reduced particularly in the case of applying insulin detemir [11,12,13,14]. The beneficial effect of insulin detemir on body weight may be stimulated by the activity of regulatory molecules, leptin and adiponectin, influencing the feeling of satiety [12,15].

Taking into account the factors mentioned above, the paramount aim of the paper is the quantitative assessment of selected regulatory molecules expression, such as leptin, leptin receptor, and adiponectin in the blood of sick, obese individuals with type 2 diabetes both before treatment, as well as six months after the pharmacological therapy with a long-lasting insulin analogue, insulin detemir. The key element is also the discriminant analysis which allows determining which biochemical or anthropometric variables discriminate the patients best before and after treatment which, at the same time, is the confirmation of the pharmacological therapy efficacy. Moreover, the relationship between the plasma concentration of both adiponectin and leptin receptor and the glycated haemoglobin value and body mass index, with the influence of gender and the time of the treatment application on the values of the examined parameters, has also been assessed.

## 2. Results

### 2.1. Biochemical Results

**Figure 1:**
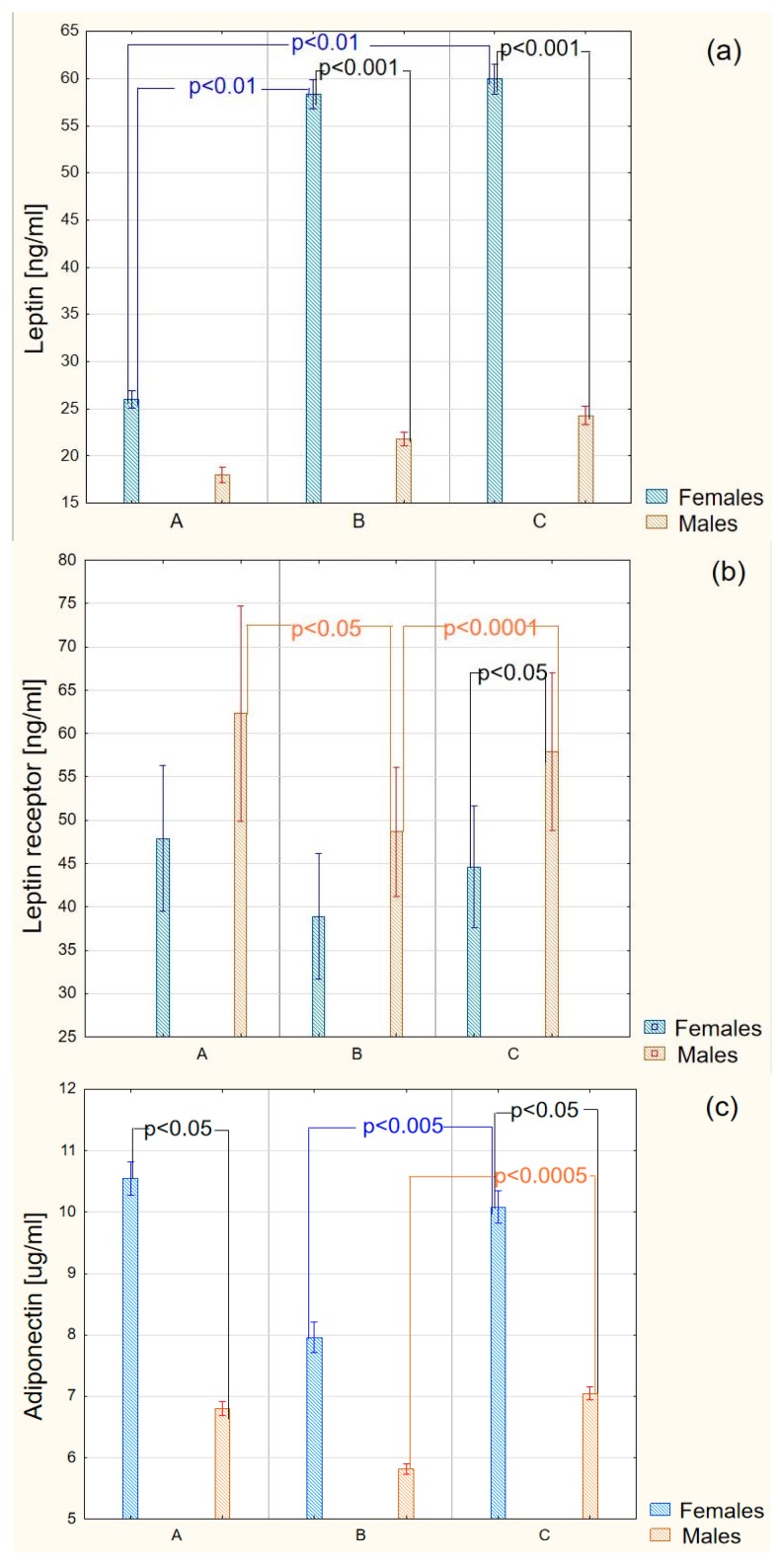
Differences in the concentration of plasma leptin (**a**), plasma leptin receptor (**b**), and plasma adiponectin (**c**) between healthy subjects (**A**) (*n* = 27), diabetic patients before treatment (**B**) (*n* = 40), and diabetic patients after a six-month therapy (**C**) (*n* = 40), depending on the gender.

The obtained results indicated that, in the blood plasma of patients suffering from diabetes, both the untreated and the treated, leptin occurs in significantly higher concentrations in relation to the concentration of this protein in the blood plasma of the individuals from the control group. Gender appeared to be an important factor modifying the leptin concentration in the blood plasma in all patients with type 2 diabetes. Significantly higher values were observed in women rather than in men. The applied therapy did not affect the plasma concentration of the studied adipokine both in the whole group and in the group divided by gender (Figure 1a).

The analysis of the relationship between the leptin concentration and Body Mass Index (BMI) revealed a positive correlation between these parameters in the group of the untreated patients (r = 0.56; *p* < 0.0005), as well those subject to the therapy (r = 0.45; *p* < 0.0005). Such a dependency was not found between the leptin concentration and the mean value of the glycated haemoglobin in the blood of patients suffering from diabetes.

Moreover, the obtained results also indicated a statistically significant difference in the concentration of the soluble leptin receptor between the group of sick individuals with diabetes, who were not subject to the therapy, and the subjects from the control group. The concentration of leptin receptor in individuals with diabetes before treatment was remarkably lower than in healthy individuals. The applied therapy caused in fact a significant increase in the plasma concentration of the analysed molecule. Unlike leptin, the plasma concentration of leptin receptor was varying in the group of men, rather than in women (Figure 1b). In the group of men suffering from diabetes, in the untreated subjects, the plasma concentration of leptin receptor was remarkably lower when compared to the concentrations observed both in the group of healthy and diabetic men after applying the therapy. The statistical evaluation of the obtained results allowed finding a negative correlation between the concentration of leptin and the concentration of leptin receptor in the blood plasma of the untreated individuals with diabetes (r = −0.55; *p* < 0.0005) and in the blood plasma of individuals with diabetes after a six-month therapy (r = −0.53; *p* < 0.0005). The analysis of the relationship between the concentration of the soluble leptin receptor and BMI indicated a negative correlation between these parameters in the group of the untreated patients (r = −0.60; *p* < 0.00005), as well as those subject to the therapy (r = −0.45; *p* < 0.005). Moreover, a significant dependence between the leptin receptor concentration and the mean value of Glycated hemoglobin (HbA1C) was found (r = −0.32; *p* < 0.05).

The results of the adiponectin plasma concentration determination indicated a significant reduction in the amount of this adipokine in patients with diabetes before treatment compared to the healthy individuals. The applied therapy resulted in an increase of adiponectin concentration in relation to the observed values in the control group. Gender appeared to be an important factor determining the concentration of this adipokine in the control group, as well as in the individuals after application of the insulin therapy (Figure 1c). The concentration of the adipokine in question was remarkably higher in women than in men in the mentioned groups. However, no relationship was found between the concentration of adiponectin and leptin, adiponectin and BMI, and adiponectin and HbA1C in the untreated patients, as well as in the patients after a six-month therapy.

### 2.2. Bioinformatic Results

#### 2.2.1. Introductory Results

The mean values of the particular measured parameters ranging from *w*(1) to *w*(21) both for the control group (written further with C in the superscript—e.g., *w^C^*(1)—as well as in the group of patients before the drug administration (marked further with G0 in the superscript—e.g., *w^G^*^0^(1)—and after six months of the therapy (marked further with G180 in the superscript—e.g., *w^G^*^180^(1). Moreover, the mean values for the difference between the values of particular parameters before and after the drug administration were also calculated, i.e., for the parameter *w*(1):

∆*w*(1) = *w^G^*^180^(1) − *w^G^*^0^(1) (1)

The obtained results are presented in Table 1. As it can be concluded from Table 1, the highest values of the mean differences of the analysed parameters in the individuals before and after the drug administration were observed for the insulin sensitivity index (%S), low-density lipoprotein (LDL) cholesterol, and the total cholesterol depicting the most pronounced influence of the treatment with insulin detemir on the metabolic control indices of patients with diabetes.

**Figure 2:**
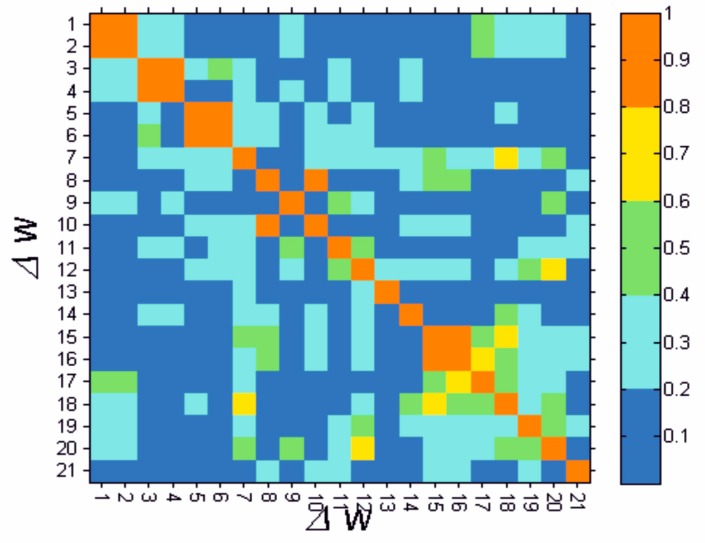
The matrix of correlation (the absolute values) for the difference of features from Δ*w*(1) to Δ*w*(21). The colour palette was accepted for determining the values of correlation. Blue, a feeble correlation; turquoise, a weak correlation; green, a moderate correlation; yellow, a strong correlation; and red, almost a complete correlation.

In the next stage of the preliminary data analysis, the correlation between the particular parameters ranging from Δ*w*(1) to Δ*w*(21) were analysed. The obtained results are shown in Figure 2.

They confirm almost a complete correlation for the difference of the features Δ*w*(1) and Δ*w*(2) (body weight and BMI), Δ*w*(4) and Δ*w*(3) (waist and hips circumference), Δ*w*(5) and Δ*w*(6) (WHR with age and WHR without age), Δ*w*(8) and Δ*w*(10) (total cholesterol and LDL cholesterol), Δ*w*(15) and Δ*w*(16) (insulin and HOMA-IR), and a high correlation for Δ*w*(7) and Δ*w*(18) (HbA1C and %B), Δ*w*(12) and Δ*w*(20) (creatinine in urine for leptin), Δ*w*(15) and Δ*w*(18) (insulin and %B), Δ*w*(16) and Δ*w*(17) (HOMA and %S). The obtained values of correlation result mainly from the natural ties between BMI and weight, waist and hips circumference, WHR indices, insulin and HOMA-IR, insulin and function index of the pancreatic islets β cells, as well as HOMA-IR and the insulin sensitivity index. On the basis of these preliminary results a further, discriminant analysis was conducted which allowed the selection of the parameters undergoing the most significant quantitative changes during the six-month therapy with insulin detemir.

#### 2.2.2. Results of Discriminant Analysis

Discriminant analysis was conducted for all the parameters from Δ*w*(1) to Δ*w*(21). The following three types of the discriminant analysis were applied: linear, fitting a multivariate normal density to each group, with a pooled estimate of covariance; diaglinear, with a diagonal covariance matrix estimate (naive Bayes classifiers); quadratic, fitting multivariate normal densities with covariance estimates stratified by group; diagquadratic, with a diagonal covariance matrix estimate (naive Bayes classifiers); and mahalanobis, using Mahalanobis distances with stratified covariance estimates.

In total 5*(2^21^ − 1) = 10,485,755 classifications were performed. Due to a relatively small number of patients (40) the number of the simultaneously-analysed parameters *w* was limited to 10 [16]. Using data mining and machine learning (e.g., discriminant analysis and others) requires the implementation of a profiled algorithm in two phases. The learning phase is based on indicating, together with the expert of cases distribution most frequently in relation to two classes (e.g., best differentiating the group of patients with type 2 diabetes before the implementation of insulin therapy, as well as after a six-month period of using insulin). The testing phase includes the comparison of learning outcomes and collected data concerning sensitivity, specificity, and accuracy of the method developed. The group of the studied individuals (40) was divided into a learning dataset and a test set, respectively, with ratios of 2/3 (27 patients) and 1/3 (13 patients).

The following were evaluated: accuracy (ACC) defined as (*TN* + *TP*)/(*FN* + *FP* + *TN* + *TP*), where *TN* represents true negative, *TP* represents true positive, *FN* represents false negative, and *FP* represents false positive. The obtained results of the first ten highest values of accuracy for the linear discriminant analysis for different configuration of Δ*w* parameters are, for example, *w*(7,8,9,10,11,13,16,21) − *ACC* = 81, *w*(4,7,8,9,10,11,20,21) for *ACC* = 81, etc.

Results for the discriminant analysis of the quadratic type are, for example, *w*(6,7,8,9,10,11,16,17) or *w*(5,7,8,9,10,11,16,17). In this case the values of ACC are at the level of 89% and 88%.

The outcomes of accuracy, for example, for the first-highest value types of discriminatory analysis is: linear ACC = 57%, diaglinear ACC = 55%, quadratic ACC = 59%, diagquadratic ACC = 59%, mahalonobis ACC = 57% for parameters Δw(6), Δw(7), Δw(8), Δw(9), Δ*w*(10), Δ*w*(11), Δ*w*(16), and Δ*w*(17).

**Figure 3:**
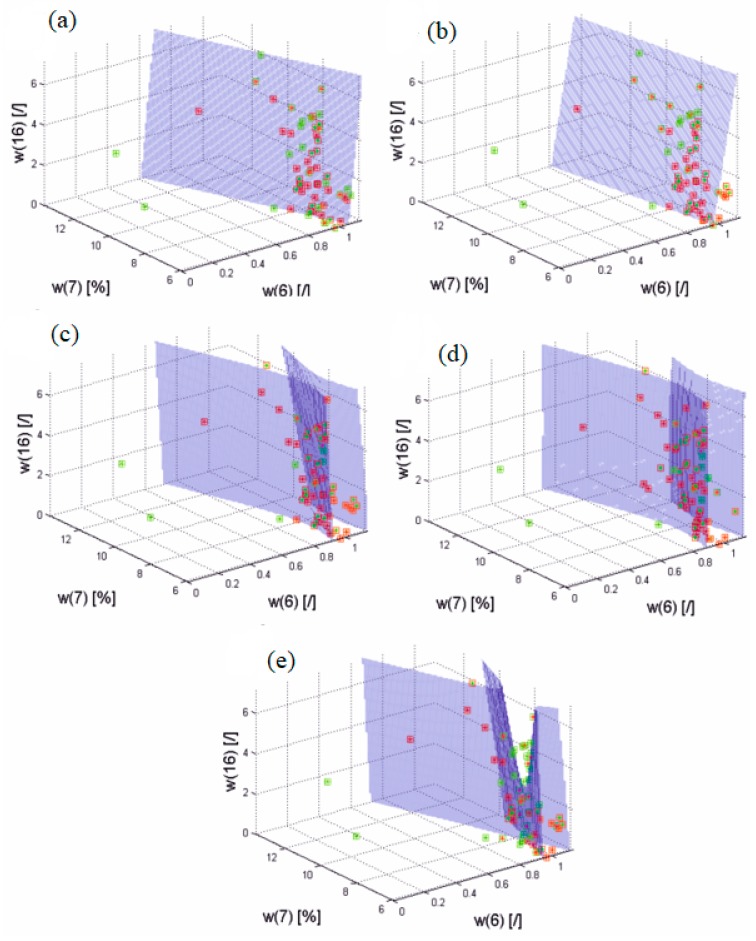
Diagrams of changes of parameters Δ*w*(6), Δ*w*(7) and Δ*w*(16) together with discriminant functions for five considered cases: (**a**) linear, (**b**) diaglinear, (**c**) quadratic, (**d**) diagquadratic, and (**e**) mahalonobis. Red marks the values of parameters before pharmacological treatment, while green marks the period of six months after treatment. A green star with a red border, and vice versa, mark false cases (false positive and false negative, respectively).

The correlation of the parameters allowing to obtain such a result is typical and concerns Δ*w*(6), Δ*w*(7), Δ*w*(8), Δ*w*(9), Δ*w*(10), Δ*w*(11), Δ*w*(16), Δ*w*(17), and in some cases Δ*w*(15) and Δ*w*(3). For Δ*w*(6), Δ*w*(7), and Δ*w*(16) selected from the mentioned parameters, a discriminant function was presented for all five considered cases, i.e., linear, diaglinear, quadratic, diagquadratic, and mahalonobis, for the test group (Figure 3).

**Figure 4:**
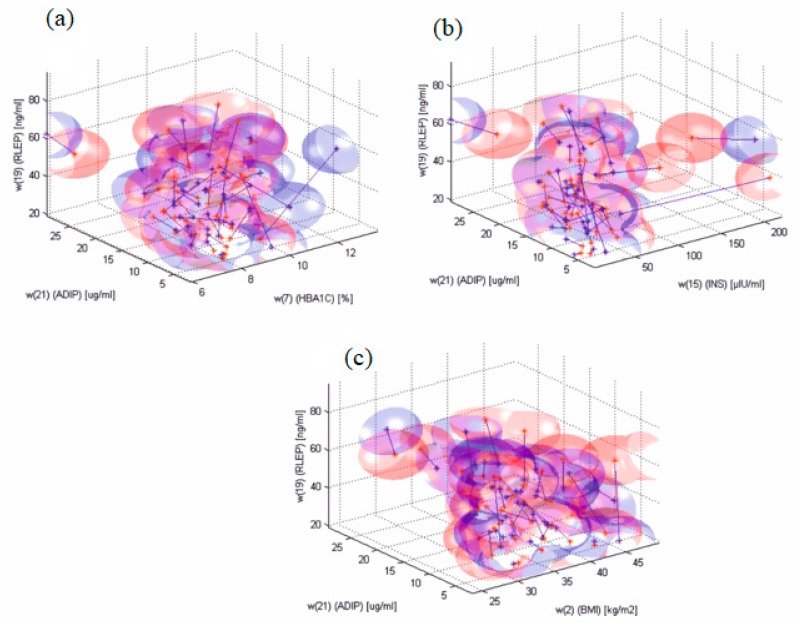
Diagrams of changes of parameters Δ*w*(19) in the function of Δ*w*(21) and (**a**) Δ*w*(7), (**b**) Δ*w*(15), and (**c**) Δ*w*(2) obtained before (blue colour) and after pharmacological treatment (red colour). Additionally, in the diagram there are lines connecting particular features for consecutive patients and also a deviation of the mean value in the form of a three-dimensional ball.

From the diagnostic point of view, the most important results of the discriminant analysis allowing the selection of the parameters best differentiating the group of patients with type 2 diabetes before the implementation of the insulin therapy, as well as after a six-month period of using insulin, were obtained for the two analysed regulatory molecules such as leptin receptor, Δ*w*(19), and adiponectin, Δ*w*(21). Among the other analysed biochemical and anthropometric parameters, the applied treatment caused the most significant quantitative changes of glycated haemoglobin Δ*w*(7), insulin, Δ*w*(15), and BMI, Δ*w*(2). Figure 4 shows diagrams of changing parameters, Δ*w*(19), in the function Δ*w*(21), while Figure 4a Δ*w*(7), Figure 4b Δ*w*(15), and Figure 4c Δ*w*(2) were obtained before and after the pharmacological treatment.

## 3. Discussion

Obesity, which accompanies type 2 diabetes, is a multifactorial disease entity being characterized by an excessive accumulation of fat tissue in the body [17]. The latter is not only treated as an “energy depot” of the organism, but mainly as an active organ of inner secretion which modifies numerous metabolic processes [18]. This tissue is a significant source of pro-, as well as anti-inflammatory factors associated with the course of the inflammatory process, i.e., adipocytokines. The numerous and complex functions of these molecules in the organism results in linkages between the fat tissue and metabolic disorders in the course of many diseases, including diabetes [19]. A significant role in regulating the adipocytokines’ expression seems to be played by exogenous insulins which are a vital element in the pharmacotherapy of diabetes [20,21]. Thus, the aim of the present paper is the assessment of the influence of the long-acting insulin analogue, i.e., insulin detemir, on the expression of the mentioned proteins in the blood of obese individuals with type 2 diabetes both before the treatment, as well as after six months of therapy with the mentioned, long-acting insulin analogue. Such a study has not been a subject of any research in the population of Polish diabetics yet.

The obtained results indicate that in the blood plasma of diabetics, both before and after treatment, leptin occurs in a significantly higher concentration than in the blood plasma of healthy individuals. Moreover, the applied treatment does not affect the plasma concentration of the protein in question. These results only partially correspond with the results described by Uribarri et al. [22]. The latter results, however, found both a significantly higher concentration of leptin in diabetics, as well as the phenomenon of the influence of the applied treatment on the plasma concentration of the mentioned protein being demonstrated by the decrease of the concentration of this adipocytokine without taking into consideration the division of the sick individuals by gender. The studies concerning the influence of insulin detemir on the plasma leptin concentration in sick individuals, albeit with type 1 diabetes, were conducted by Zachariah et al. [23]. However, they concerned only the effects of using two types of insulin, i.e., insulin detemir and NPH insulin without taking into consideration the control group (healthy individuals) or a possible influence of the applied treatment on the plasma leptin concentration. The studies revealed a decrease in the leptin concentration in the blood of patients treated with insulin detemir in relation to the concentration of this protein in the sick individuals’ blood treated with NPH insulin. It is difficult to compare the study results described by Zachariah et al. [23] with the obtained ones in this paper as there are two different scientific assumptions of both experiments, a different selection of the studied groups and, not least, a different type of diabetes from which the patients covered by the study were suffering from. It is likely that a different character of changes in relation to the obtained ones in this paper may also be linked to the pathomechanisms dissimilarity of both types of the disease. The results in this paper prove the lack of changes of leptin concentration after six months of the therapy with a long-acting analogue, insulin detemir. They correspond with Gurkan et al.’s study results [24] who, after 26 weeks of the therapy with a different long-acting analogue, insulin glargine, did not observe the increase in the leptin concentration in the blood plasma [24]. Similar study results were described by Bunck et al. [25] who, after a 12-month therapeutic period with insulin glargine, did not confirm the increase in the leptin concentration in the plasma of sick individuals with type 2 diabetes. However, Joy-Galean et al.’s observations [26] indicate an increase in the leptin concentration in patients with type 2 diabetes after a six-month treatment with insulins glargine and apidra. A different tendency of the latter in relation to the above-mentioned may be linked to a different model of therapeutic approach accepted by the authors of the paper.

In this paper it has also been demonstrated that the leptin concentrations in the blood of women with diabetes were significantly higher than in men. The results are concurrent with the observations of other authors who stated that the plasma leptin concentration in women is two or three times higher than the concentration in the plasma of men with the same BMI as in women, both in the case of healthy individuals, as well as those with obesity [27]. A similar tendency was also described by Ogawa et al. [28] who demonstrated a statistically significant increase in the concentration of the mentioned protein in the blood of women with type 2 diabetes in relation to the value of the leptin concentration found in sick men. The observed differences in the profile of leptin concentration in the blood of obese people with type 2 diabetes, depending on the examined individuals’ gender, should be, on one hand, linked with a greater secretory function of the fat tissue in women in relation to leptin and adiponectin, while, on the other hand, with a different impact of sexual hormones on secreting the adipokine in question [29].

Moreover, in the present paper a positive high correlation has been indicated between the leptin concentration in the blood plasma of diabetics, both the untreated and those subject to insulin therapy, and the body mass index of the studied individuals. Similar relationships were described in obese individuals with type 2 diabetes and without diabetes, as well as non-obese with diabetes [30,31,32,33]. The obtained results seem to correspond with that described by other authors; the phenomenon of the plasma leptin concentration relationship with the level of obesity, the volume of body fat, insulin resistance, plasma concentration of triacylglycerols, and pro-inflammatory cytokines [34]. The conducted studies did not indicate a relationship between the leptin concentration and the value of the glycated haemoglobin percentage in the blood of the individuals suffering from diabetes both before and after insulin therapy. The obtained results mostly correspond with the ones described by Vinitha et al. [34], Mohiti et al. [35], Owecki et al. [36], and Taghdir et al. [37] who did not find a statistically significant dependence between the parameters in question in sick individuals with type 2 diabetes or with an impaired glucose tolerance, although a reverse correlation between the leptin concentration and the value of HbA1c in individuals with the mentioned diseases was also mentioned [38]. The non-equivalent tendency of the relationship, in the mentioned results, between the studied parameters may result from a different level of glycaemic control in the examined patients, as well as from a distinct methodology of the applied biochemical determinations in the paper by Moriya et al. [38].

The subject of the present study was also the evaluation of the soluble leptin receptor concentration (OB-Re or sOB-R) in sick individuals’ blood. There are five isoforms of this receptor, i.e., OB-Ra, OB-Rb, OB-Rc, Od-Rd, and OB-Re or, more often, sOB-R. The first four are membrane isoforms, while the last one, described as “e” (or sOB-R) is a soluble receptor determined also as secretory, not related with cytoplasm or cell membranes [39,40]. The soluble form of the receptor for leptin may play a key role in regulating leptin concentration in blood. It has been demonstrated that under conditions with a smaller amount of body fat tissue, the leptin concentration in blood is reduced, while the expression of the gene coding the soluble isoform of the leptin receptor intensifies [41]. Moreover, it has also been confirmed that the soluble form of the leptin receptor conditions/modulates the bioavailability, as well as hormone tissue functioning, inhibits binding of leptin with membrane receptors, and stunts the clearance of the protein in question [42].

The obtained results indicate a lower concentration of this receptor in the blood plasma of the untreated patients with diabetes in relation to healthy individuals. The therapy caused a significant increase in the concentration of the studied molecule to the value of the observed individuals from the control group. The studies seem to reflect a potentially beneficial influence of the applied treatment. The thesis may be confirmed by the results described by Ogawa et al. [28], who found the phenomenon of a negative correlation between the concentration of the soluble leptin receptor in the blood and the intensified level of insulin resistance.

In the analysis of the influence of gender on the plasma concentration of the mentioned receptor in particular study groups, a significant difference was found between women and men only in the case of patients suffering from diabetes after the applied treatment. The concentration of the plasma leptin receptor in women was remarkably lower in women than in men. The tendency of the latter changes is partially compatible with the one described by Ogawa et al. [28], who confirmed statistically significant differences between the concentration of the soluble receptor in men and in women, both the healthy and those suffering from type 2 diabetes, where the concentration of the protein in question was always remarkably higher in men’s organisms.

It should be mentioned that the biological role of the soluble receptor for leptin was not fully explained. However, as it has been mentioned above, the protein delays the leptin clearance from circulation, which increases the biologic availability of the mentioned adipokine and intensifies its action [43]. The obtained results in this paper pointed out a high negative correlation between the concentration of the mentioned receptor and the concentration of its ligand, leptin, in the blood plasma of diabetics both before treatment and after application of the therapy. These results were confirmed in Ogawa et al. observations [28] who found a negative correlation between the plasma concentration of sOB-R and the leptin concentration. The results of the conducted analyses also indicated both a high negative dependence between the leptin receptor concentration in the blood plasma of patients with diabetes—the untreated and their BMI, as well as an averagely negative correlation between the leptin receptor concentration in the blood plasma of diabetics after treatment and their body weight. The presented tendency of these changes correspond with the character of the changes described by Laimer et al. [44] and Van Dielen et al. [45] who proved that the loss of body weight of obese people leads to an increase in the plasma concentration of the soluble leptin receptor. As it is known, the plasma concentration of the receptor in question is one of the factors influencing the level of insulin resistance and leptin resistance [28]. The latter phenomenon is accompanied with an increased fat tissue in the body and an increase in the leptin concentration in the blood [46].

The studies conducted in this paper also indicated an average, negative correlation between the concentration of the soluble leptin receptor and the value of the glycated haemoglobin in the blood of people with newly-diagnosed type 2 diabetes. This is compatible with the results obtained by other authors, however, for patients with type 1 diabetes [47].

The aim of the present paper was also the evaluation of the concentration of the other regulatory molecule, adiponectin, in the blood of obese patients with type 2 diabetes. A special property of this cytokine is the ability to “insulin-sensitizing” performed by the binding of this adipokine to its receptors: AdipoR1 and AdipoR2 [4]. This characteristic is reflected by an intensification—in the skeletal muscles—of the expression of the molecules transporting the fatty acids, such as CD36 and the oxidase of the acyl-coenzyme A, also involved in utilizing fatty acids and uncoupling of UC-2 protein, which is necessary in the course of the energy expenditure process [48,49].

The conducted quantitative assessment of adiponectin indicated a significantly lower concentration of this adipocytokine in the blood plasma in individuals suffering from diabetes—before treatment, in relation to its concentration in the healthy individuals’ blood plasma as well as in those subject to insulin therapy. The results are in agreement with the results described by other authors [22,26]. Moreover, the dose of insulin detemir applied in this paper caused an increase in the concentration of this adipokine both in men and in women, reflecting the beneficial influence of the therapy with the applied insulin analogue on the impaired systemic metabolism in the course of diabetes.

In the group of healthy women and those suffering from diabetes subject to therapy, the concentrations of adiponectin were higher than in men, which is compatible with the results of Ogawa et al. [28] and Aleidi et al. [50]. The gender-associated differences in the plasma concentration of the adipocytokine in question may result from a higher secretory function of the fat tissue in women, as well as the character of sexual hormone activity against the biosynthesis and secretion of this protein [29]. The described adipocytokine, together with leptin, which is the second key adipocytokine, take part in the regulation of glucose metabolism and tissue sensitivity to insulin [7]. Taking into consideration the fact that the presented studies indicated the metabolism impairment of both adipocytokine in obese individuals with type 2 diabetes, the assessment concerned the profile of the correlation between these two regulatory molecules. The conducted studies did not indicate the relationship between the concentration of leptin and adiponectin in the examined sick individuals, both those with newly-diagnosed diabetes and those after implementation of insulin therapy. The lack of the relationship between the two adipocytokines in sick individuals with type 2 diabetes was also found in the studies by Jung et al. [51], however, on the other hand, a negative correlation between both adipocytokines in type 2 diabetics was described by Al-Hamodi et al. [52]. It is difficult to explain the discrepancy between the presented results, particularly the lack of the relationship between the concentration profile of both adipocytokines, which appears to contradict the tendencies of their serous concentration described in this paper, where the lowering of the concentration of one adipocytokine (adiponectin), in the course of diabetes, was accompanied by the increase in the concentration of the second one (leptin).

The conducted studies revealed a lack of correlation between the plasma concentration of adiponectin and the body mass index in patients with type 2 diabetes. There was also no demonstration of any relationship of the plasma concentration of adiponectin with the level of the metabolic control of diabetes, which corresponded with the results described by Schulz et al. [53] and Vinitha et al. [34].

## 4. Material and Methods

### 4.1. Subjects

The material in the study was constituted by blood samples collected from 40 obese people, of both sexes, between 44 and 72 years of age, with diagnosed type 2 diabetes. The control material in the study was comprised of blood samples collected from 27 healthy individuals, of both sexes, between 40 and 67 years of age. The study was approved by the Bioethics Committee of the Medical University of Silesia in Katowice (KNW/0022/KB1/147/10). The assessment of the anthropometric parameters and laboratory biochemical examinations in patients diagnosed with type 2 diabetes was performed twice: before the beginning of the treatment and after six months of the therapy with a long-acting human insulin analogue—insulin detemir—administered once a day, in the evening in doses from 0.2 unit/kg b.w. or 10 units/day [54].

The inclusion criteria referred to obese male and female patients of all ethnic groups with recognized type 2 diabetes, treated with metformin; patients who reached the age of 18 years and could decide themselves about participating in the study; patients who were able to understand the aim and possible risk associated with participation in the study after signing the informed consent for the study and expressing their consent. The exclusion criteria: patients with type 1 diabetes; patients with different types of diabetes; female patients with gestational diabetes and diabetes during lactation; patients with autoimmune diseases; patients taking glycocorticosteroids, Adrenocorticotropic hormone (ACTH), and interferons; patients with prior stroke or myocardial infarction; patients with unstable angina; patients with class III and IV class heart failures according to New York Heart Association (NYHA); patients with kidney failure; patients with liver failure. The results of morphological and biochemical blood analyses of healthy subjects enrolled to this study (such as electrolytes, fasting glucose, fasting lipid profile, creatinine, bilirubin, protein electrophoretic profile, and C-reactive protein (CRP)) fell within the reference range.

### 4.2. Biochemical Analysis

In each sick individual an interview and physical examination were performed. In the interview the following data of patients were collected: age, sex, and disease duration. The physical examination comprised of the measurement of body weight, *w*(1), based on which the Body Mass Index was calculated, *w*(2), as well as the measurement of the waist *w*(3) and hips, *w*(4), based on which the waist/hips ration (WHR) was calculated. The WHR index considered age, *w*(5), the average for the population considering age, and the WHR index without any reference to age, *w*(6). The systolic and diastolic arterial blood pressures were also measured.

A series of laboratory tests was also performed in each patient, which was used to monitor the diabetic patients. The tests included the value of glycated haemoglobin, *w*(7), the concentration of total cholesterol, *w*(8), HDL cholesterol, *w*(9), Low-density lipoprotein (LDL) cholesterol, *w*(10), and triacylglycerols, *w*(11), creatinine, *w*(12), albumin in urine, *w*(13), glucose concentration, *w*(14), and insulin, *w*(15). Additionally, the value of the HOMA-IR insulin-resistance index was also determined, *w*(16), insulin sensitivity index (%S), *w*(17), and the function index of the pancreatic islet β cells (%B), *w*(18), as well as the leptin receptor concentration in blood plasma, *w*(19), leptin, *w*(20), and adiponectin, *w*(21).

The concentration assessment in the blood plasma of both healthy individuals and patients suffering from type 2 diabetes involved the following tests based on the enzyme-linked immunosorbent assay (ELISA): the test of DRG Instruments GmbH, Marburg, Germany for leptin; the test of Mediagnost GmbH, Reutlingen, Germany for adiponectin, and the test of BioVendor R and D, Brno, Czech Republic for leptin receptor.

### 4.3. Statistical Analysis

The obtained results underwent a statistical calculations with the use of the computer program STATISTICA of StatSoft, Inc. (2011) (StatSoft, Cracow, Lesser Poland, Poland), (data analysis software system) version 10 (www.statsoft.com). The statistical analysis covered testing the significance of the mean value differences of the given characteristic for the control group and the study group with the use of Student’s *t*-test for independent samples, while in the case of the group of the studied patients before administering the drug and the group of patients after a six-month pharmacotherapy—with the use of Student’s *t*-test for continuous variables. The same statistical significance level *p* < 0.05 was accepted for all applied tests and statistical analyses. The correlation strengths of two variables was assessed with the Pearson correlation r coefficient.

### 4.4. Bioinformatic Analysis

The data analysis was conducted with the use of Matlab with the Signal Processing Toolbox (Matlab: Version 7.11.0.584, R2010b, Java VM Version: Java 1.6.0_17-b04 with Sun Microsystems Inc. (MathWorks®, Natick, MA, USA); Signal Processing Toolbox: Version 7.1) on a PC running Windows 7 Professional, 64-bit with an Intel Core i7-4960X CPU @ 3.60 GHz (MathWorks®, Natick, MA, USA).

The bioinformatic data analysis was performed in two stages:

The first stage included a preliminary data analysis: the calculations of the mean values of differences between particular parameters, standard deviations of the mean and the matrix of correlation between all the measured parameters;

The second stage covered the selection of the most significant diagnostic features, proposing a discriminant analysis which allows selecting the variables which best forecast the efficacy of the therapy [16,55,56].

## 5. Conclusions

Summing up, the conducted studies have indicated the participation of adipocytokines—the biologically-active mediators of the systemic metabolism—such as leptin and adiponectin, in the pathomechanisms of disorders underlying obesity which, in turn, leads to the development of insulin resistance and consequently contributes to the occurrence of type 2 diabetes. The conducted discriminant analysis has also demonstrated that the concentration of the soluble leptin receptor and adiponectin best reflect the influence of the applied therapy on the endocrine activity of the fat tissue in the course of type 2 diabetes. The complement of the knowledge concerning the regulatory mechanisms influencing the activity of adipocytokines participating in controlling the feeling of appetite and satiety, as well as energy management, may enable the introduction of new therapeutic strategies based on a targeted and more effective therapy of disorders occurring with energy efficiency disorders, such as obesity or type 2 diabetes.

## Figures and Tables

**Table 1:** Clinical and anthropometric parameters of the healthy subjects and diabetic patients before and after the six-month insulin therapy.

		Healthy Subjects A	Untreated Diabetic Patients B	Diabetic Patients after Six-Months of Therapy C
Weight (kg)	*w*(1)	84.4 ± 22.4	94.9 ± 14.6 ^a^	95.2 ± 15.4 ^a^
BMI (kg/m^2^)	*w*(2)	30.63 ± 5.4	34.18 ± 5.43 ^b^	34.4 ± 5.5 ^b^
Waist (cm)	*w*(3)	100.0 ± 15.3	112.7 ± 11.4 ^b^	111.2 ± 10.2 ^b^
Hips (cm)	*w*(4)	105.6 ± 15.9	112.6 ± 11.4 ^a^	111.5 ± 10.6
Waist-hip ratio (WHR) (cm^2^)	*w*(5)	0.95 ± 0,1	0.9995 ± 0.049	1.001 ± 0.06
WHR without any reference (cm^2^)	*w*(6)	0.95 ± 0,1	0.9995 ± 0.049	1.003 ± 0.06
Glycated hemoglobin (%)	*w*(7)	5.17 ± 0.45	7.87 ± 1.4 ^b^	7.78 ± 0.89 ^b^
Total cholesterol (mg/dL)	*w*(8)	192.3 ± 34.5	196.4 ± 36.1	188.8 ± 30.3
High-density lipoprotein (HDL) cholesterol (mg/dL)	*w*(9)	46.8 ± 8.76	49.5 ± 13.6	48.1 ± 12.9
LDL cholesterol (mg/dL)	*w*(10)	115.7 ± 28.6	111.4 ± 32.9	99.1 ± 29.9
Triacylglycerols (mg/dL)	*w*(11)	145.7 ± 60.7	176.8 ± 88.4	182.5 ± 68.5
Creatinine (mg/dL)	*w*(12)	0.84 ± 0.09	0.834 ± 0.528	0.993 ± 0.86
Albumin in urine (µg/mL)	*w*(13)	2.5 (2.5–4.3) *	4.3 (2.5–15.50) *^,a^	2.5 (2.5–8.4) *
Glucose (mg/dL)	*w*(14)	90.85 ± 7.3	155.3 ± 45.2	150.6 ± 33.01
Insulin (µIU/mL)	*w*(15)	10.6 (4.7–23.8) *	11.4 (4.5–25.6) *	16.0 (7.6–29.9) *^,a^
homeostatic model assessment-insulin resistance index (HOMA—IR)	*w*(16)	1.29 (0.5–2.4) *	2.0 (1.2–3.9) *^,a^	2.5 (1.6–3.8) *^,a^
Insulin sensitivity index (%S)	*w*(17)	77.6 (41.1–189.5) *	50.7 (25.8–86.8) *^,a^	40.2 (26.4–63.9) *^,b,c^
Function index of the pancreatic islets β cells (%B)	*w*(18)	98.8 (70.6–171.0) *	53.5 (32.0–92.9) *^,b^	71.9 (41.6–92.2) *^,b^
Leptin receptor (ng/mL)	*w*(19)	56.9 ± 21.4	44.3 ± 16.4 ^b^	51.9 ± 18.9 ^d^
Leptin (ng/mL)	*w*(20)	21.1 ± 10.8	39.0 ± 16.2 ^a^	39.6 ± 18.4 ^a^
Adiponectin (µg/mL)	*w*(21)	9.01 ± 3.7	6.8 ± 4.2 ^a^	8.4 ± 4.6 ^d^

* results are expressed as the medians (quartile 1; quartile 3). Statistically significant differences (^a^ *p* < 0.05, ^b^ *p* < 0.01 compared to control group; ^c^ *p* < 0.01, ^d^ *p* < 0.001 compared to untreated patients with type 2 diabetes).
